# Removal of Pb(II) ions from lead-ammonia complex wastewater by the magnetite particles prepared at ambient temperature

**DOI:** 10.1016/j.isci.2026.115262

**Published:** 2026-03-06

**Authors:** Liu Fang, Zhou Kanggen

**Affiliations:** 1 College of Material and Energy Engineering, Lishui University, Lishui, Zhejiang 323000, China; 2 College of Metallurgical and Environmental Engineering, Central South University, Changsha, Hunan 410128, China

**Keywords:** Applied sciences, Water resources engineering

## Abstract

Magnetite (Fe_3_O_4_) were successfully synthesized at ambient temperature (25°C) as the adsorbent for the removal of Pb(II) ions from lead-ammonia wastewater. The magnetite exhibited a magnetization saturation value of 82.30 emu/g, illustrating the magnetite particles could be readily recovered from water under an external magnetic field. Comparing the removal capacity of Pb(II) ions in solution with NH_3_-N and without NH_3_-N, and several factors effecting the adsorption of Pb(II) ions such as pH, contact time and ammonia nitrogen were investigated. The adsorption of Pb(II) ions onto the magnetite fitted well with the pseudo-second-order kinetics and Langmuir isotherm model. And the maximum adsorption capacities reached up to 123.15 mg/g. Ammonia nitrogen in solution could hinder the adsorption of Pb(II) ions. This study demonstrates that magnetite offers a cost-effective solution for treating complex wastewater, with potential applications in industrial wastewater treatment for heavy metal removal.

## Highlights

• Magnetite was green-synthesized at ambient temperature and used as an adsorbent
• Effects of pH, contact time, initial Pb(II) concentration, and nitrogen on adsorption studied
• Pb(II) ion adsorption followed pseudo-second-order kinetics and Langmuir isotherm model
• NH_3_-N in this system could inhibit Pb(II) adsorption and was also removed

## Graphical abstract

**Figure:**
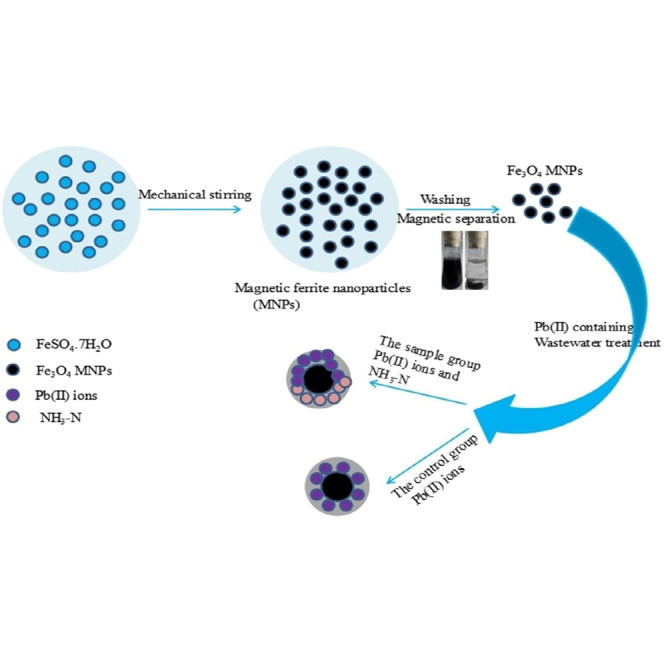

## Introduction

Industrial wastewater often contains a complex mixture of heavy metals and other pollutants, with lead-ammonia wastewater being a particularly common example. Such wastewater typically originates from anthropogenic activities, including electroplating, storage battery manufacturing, fertilizer production, metallurgy, and mining.^1^^,^^2^ Unlike traditional organic pollutants or phosphorus, lead-containing wastewater poses a significant environmental threat due to its non-biodegradability and bioaccumulative nature. Lead can accumulate through food chains, affecting higher trophic levels and causing adverse effects on various organisms.^3^^,^^4^ It also leads to severe ecological problems.^5^^,^^6^ Excessive lead exposure in humans can result in a range of health disorders, including anemia, immune system dysfunction, cumulative poisoning, kidney damage, and nervous system disorders.^7^^,^^8^ Additionally, the excessive discharge of nitrogen-rich wastewater can deteriorate groundwater quality and cause eutrophication, further complicating the removal of coexisting lead in the aqueous environment.^9^ Therefore, it is crucial to develop effective and economical technologies to remove lead from complex wastewater before it is discharged into the environment.

Various conventional technologies have been employed to remove lead ions or nitrogen from industrial effluents. The main treatment methods include chemical precipitation,^10^ membrane separation,^11^ reverse osmosis,^12^ adsorption,^13^ ion exchange,^14^ and electrolysis.^15^ However, these approaches often face economic or technical challenges, such as high costs, complex operations, significant energy consumption, difficulties in separating sludge from water, secondary pollution, or the generation of toxic sludge.^16^^,^^17^ In recent years, adsorption has emerged as a preferred technique due to its high efficiency, ease of operation, environmental friendliness, applicability at low concentrations, and cost-effectiveness.^18^^,^^19^^,^^20^ The success of adsorption largely depends on the adsorbent’s properties, such as high surface area and sufficient active sites.^21^^,^^22^

Over the years, numerous adsorbents have been developed and tested for the removal of metal ions from complex wastewater. These include activated carbon,^23^^,^^24^ ion-exchange resins,^25^^,^^26^ and inorganic minerals.^27^ Recently, magnetite particles have garnered significant attention in environmental remediation. The magnetite nanoparticles (MNs) are chosen as an adsorbent due to their cost-effectiveness, chemical stability, ease of synthesis, low toxicity, and high adsorption capacity (Table 1). Most importantly, their unique magnetic properties allow for easy separation from the aqueous system using an external magnetic field.^28^^,^^29^^,^^30^ Recent advancements suggest that MNs can effectively address many environmental issues in wastewater. However, few studies have explored the removal of Pb(II) ions from complex systems using the MNs. Thus, this study has been conducted on lead-ammonia wastewater based on the MN technology.

**Table 1:** Comparison of adsorption capacities for Pb(II) on MNs versus other adsorptions

Adsorbents	Adsorption capacity (mg/g)	Reference
MNs	123.15	This study
Carbon nanotube	17.4	Li et al.^31^
Fe_3_O_4_/cyclodextrin polymer nanocomposites	64.5	Badruddoza et al.^32^
Iron oxide nanoparticles	36.0	Nassar^33^
Chitosan/magnetite nanocomposite	63.33	Tran et al.^34^
Magnetite nanospheres	13.4	Kumari et al.^35^
Fe_3_O_4_/bacterial cellulose nanocomposite	65.0	Zhu et al.^36^
Multiwalled carbon nanotubes	33.0	Zhao et al.^37^

The study highlights the green synthesis of the magnetite and investigates the feasibility of using the as-prepared magnetite as an adsorbent for the removal of Pb(II) ions from lead-ammonia wastewater. Effects of solution pH, contact time, and ammonia nitrogen in coexisted wastewater on Pb(II) ion adsorption were discussed. Moreover, the adsorption behavior, including the adsorption kinetics and isotherms, was also elucidated.

## Experimental methods

### Materials

In this study, all chemicals and reagents were of analytical grade and were sourced from Lishui Chemical Reagent Company (Zhejiang, China) without undergoing additional purification. The specific reagents utilized included ferrous sulfate (FeSO_4_·7H_2_O), sodium hydroxide (NaOH), hydrochloric acid (HCl), lead nitrate [Pb(NO_3_)_2_], EDTA, sulfosalicylic acid, hexamethylenetetramine, and dimethyl phenol orange indicator. Throughout the experimental process, double-distilled deionized water was consistently employed.

### Synthesis of the magnetite

The synthesis process was conducted at room temperature (25.0°C). Initially, 1.5 L of distilled water was added into a 2.0 L flat-bottom flask. Then it was flushed with nitrogen gas for 10 min and plugged with a rubber stopper to eliminate partly dissolved oxygen. Subsequently, 7.5 g of ferrous sulfate was introduced into the flask and thoroughly mixed using a magnetic stirrer. During this process, the pH of the solution was adjusted to 9.0 using 1.0 mol/L sodium hydroxide and 1.0 mol/L hydrochloric acid solutions. The chemical reaction for the formation of magnetite is described in Equation 1. After the reaction was complete, the resulting black mixture was collected using a magnet, washed multiple times with ethanol and water, and then dried in a vacuum oven at 60°C for further characterization and analysis.

(Equation 1) $$3{\text{Fe}}^{2+}+4{\text{OH}}^{-}+O_{2}\to {\text{Fe}}_{3}O_{4}+2H_{2}O$$

### Characterization and analysis

The characterization of the synthesized magnetite particles in this study was conducted using a variety of standard analytical techniques. The crystal structure was analyzed using an MSAL-XD2 X-ray diffractometer (XRD) with Cu Kα radiation (λ = 0.1541 nm) over a 2θ range of 10°–80°, operated at 30 kV and 30 mA. The surface composition was elucidated using X-ray photoelectron spectroscopy (XPS). The morphology and particle size were examined with a scanning electron microscope (SEM) at an electron acceleration voltage of 20 kV and a laser particle size analyzer (LPSA). The magnetic properties were assessed by a vibrating sample magnetometer (VSM). The specific surface area was measured by the Brunauer-Emmett-Teller (BET) method using N_2_ adsorption/desorption. Additionally, the Fe^2+^/Fe^3+^ ratio was determined via complexometric titration.

### Batch adsorption experiments

The effectiveness of the MNs in adsorbing Pb(II) ions was assessed through batch adsorption experiments. The stock solution of Pb(II) ions (1,000 mg/L) was prepared by dissolving Pb(NO_3_)_2_ in double-distilled water and then diluted to various concentrations ranging from 25 to 150 mg/L for use in the experiments. The experimental design included two groups of Pb(II) solutions: a control group without NH_3_-N and a sample group containing 50 mg/L NH_3_-N. In each experiment, a predetermined amount of magnetite particles was added to a 250 mL flask containing a specific concentration of Pb(II) ions. The flasks were sealed with rubber stoppers and stirred continuously during the reaction. The pH of the reaction mixture was adjusted using 0.1 M HCl and 0.1 M NaOH solutions. After the reaction, the reaction pH was adjusted using 0.1 M HCl and 0.1 M NaOH. After reaction, Pb(II) ions in supernatant solution was measured by an inductively coupled plasma-optical emission spectroscopy (ICP-OES) as the residual concentration (C_t_), and the removal rate (η) and the adsorption capacity (Q_e_) were calculated by the following equations:

(Equation 2) $$\eta =\left[\left(C_{o}-C_{e}\right)/C_{o}\right]\times 100\%$$

(Equation 3) $$Q_{e}=\left[\left(C_{o}-C_{e}\right)\times V/m\right]$$

where C_o_ and C_e_ were the initial and the equilibrium concentrations of Pb(II) ion (mg/L), respectively, V is the volume of Pb(II) ion solution (L), m is the weight of the ferrite used, and η is the removal rate of Pb(II) ion (%).

## Results and discussion

### Characterization of magnetite particles

**Figure 1:**
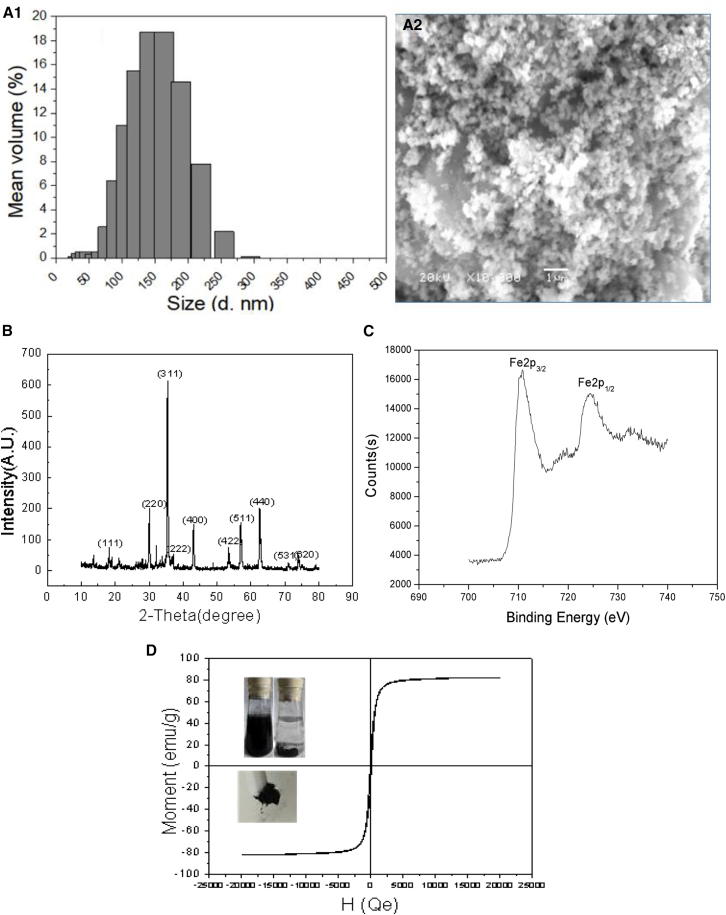
LPSA, SEM, XRD, XPS, and VSM analysis of the magnetic particles (A1) LPSA. (A2) SEM, scale bar = 1 μm. (B) XRD. (C) XPS. (D) VSM.

The particle size, morphology, crystalline structure, and magnetic performance of the as-prepared product were analyzed using LPSA, SEM, XRD, XPS, and VSM analysis, respectively. The corresponding results are shown in Figure 1.

As indicated in Figure 1, a1, the synthesized particles exhibit a size ranging from 25 to 275 nm, with an average diameter of 150 nm. The SEM image (Figure 1, a2) shows that these particles have a rough surface and are mono-dispersed, displaying a narrow size distribution that aligns with the particle size data obtained from LPSA. Additionally, N_2_ adsorption-desorption isotherms were utilized to evaluate the specific surface area and pore structure of the magnetic particles. The analysis revealed the BET surface area of 26.78 m^2^/g, a pore volume of 0.144 cm^3^/g, and an average pore size of 22.33 nm.

The crystal structure of synthesized magnetic particles was characterized by XRD, as shown in Figure 1B. The XRD patterns displayed 11 distinct reflection peaks at (111), (220), (311), (222), (400), (422), (511), (440), (531), and (620), which correspond well with the cubic inverse spinel structure of the Fe_3_O_4_, as documented in the JCPDS card no. 79-0417.^38^ This confirmed that the particles synthesized in this study were Fe_3_O_4_. XPS was employed to further detect the composition of magnetic particle (Figure 1C). The Fe2p spectrum displayed characteristic peaks at approximately 710.5 eV (Fe2p_3_/_2_) and 724.4 eV (Fe2p_1_/_2_), which were indicative of Fe_3_O_4_,^39^ and no significant peaks corresponding to Fe_2_O_3_ were observed. Thus, the survey of Fe 2p peak agree with the data for referenced magnetite,^40^ which confirmed that the particles were indeed Fe_3_O_4_ and not other iron oxides. Additionally, the Fe^2+^/Fe^3+^ ratio of the synthesized MNs was determined to be 0.5, which was consistent with that in Fe_3_O_4_, indicating that the magnetite particles were successfully synthesized.

Furthermore, Figure 1D illustrated the magnetic property of magnetite particles. The M-H curve indicates that the magnetic ferrite is essentially super-paramagnetic, with a magnetization saturation value of 82.30 emu/g. This property enables the MNs dispersed in water to be rapidly collected using an external magnetic field within just a few minutes. Then it can be readily re-dispersed with slight shake, as illustrated in the insets of Figure 1D. The findings demonstrate that the magnetite particles possess excellent magnetic responsiveness and re-dispersion capabilities, highlighting the potential of MNs as an effective adsorbent in aqueous system.

### The removal of Pb(II) ions from lead-ammonia wastewater

#### Effect of solution pH

**Figure 2:**
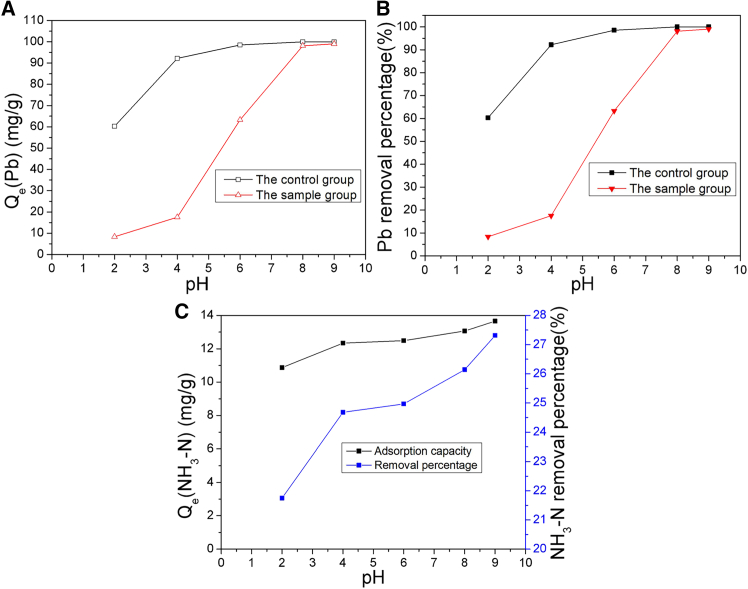
The effect of pH on adsorption of Pb(II) ions by the magnetic ferrite and the removal of NH_3_-N in the sample group (A) Qe. (B) Removal percentage. (C) NH_3_-N removal.

The solution pH plays a critical factor influencing the adsorption efficiency. In the experiments conducted on both groups, the pH was adjusted between 2.0 and 9.0 using NaOH or HCl. The adsorptions were carried out at 25°C for 60 min, and 0.2 g of MNs was mixed with 200 mL of a 100 mg/L Pb(II) ion solution in a shaker. The outcomes are depicted in Figure 2.

As illustrated in Figures 2A and 2B, the trends in Pb(II) ion adsorption capacity and removal percentage were consistent for both groups. The adsorption capacity of Pb(II) ions in the control group surged significantly as the pH value rose from 2.0 to 6.0. Beyond the point, the increase gradually slowed down and ultimately reached an equilibrium within the pH range of 6.0–9.0. As the pH was adjusted to 9.0, the removal percentage reached 99%. The sample group demonstrated a heightened responsiveness to pH variations. The adsorption ability of Pb(II) ions increased obviously as the pH rose from 2.0 to 7.0. Following this, the increase slowly rose and reached a stable point up to pH 9.0. At pH 7.0, both the adsorption capacity and removal percentage achieved impressive levels of 97.0 mg/g and 97%, respectively. Simultaneously, the magnetite particles in the sample group effectively facilitated the removal of NH_3_-N from the solution, as demonstrated in Figure 2C. Both the adsorption capacity and removal percentage of NH_3_-N demonstrated an enhancement with rising solution pH, ultimately reaching final values of 13.66 mg/g and 27.32%, respectively.

Figure 2 illustrates that the solution pH has a substantial impact on both the surface charge of magnetite particles and the type of lead ions in solution. Ozmen et al.^41^ reported that Fe^2+^ on the magnetite surface hydrolyzes into FeOH^+^ below pH_zpc_ (7.5) and forms Fe(OH)_3_^−^complexes above the pH_zpc_. Hence, in the control group, the magnetite surface was predominantly charged with positive ions, and the H^+^ competed with Pb(II) ions for adsorption sites at pH value ranging from 2.0 to 7.0,^42^ which markedly decreased Pb(II) ion adsorption, thereby diminishing its effectiveness in removing Pb(II) ions from aqueous system. With the pH rising, the magnetite surface’s charge progressively shifted toward a negative state. At pH 8.0, Pb^2+^ was the dominant species, with a slight amount of PbOH^+^ or Pb(OH)_2_, Pb(OH)_2_ emerging as the primary species at pH value exceeding 8.0.^43^ This shift amplified the electrostatic attraction between the lead ions and magnetite particles. In the sample group, the species of ammonia nitrogen (NH_4_^+^or NH_3_) were affected by both solution pH and temperature. At ambient temperature, the proportion of free NH_3_ was zero at pH 8.0 and reached approximately 25% with pH rising to 9.0. So at lower pH, NH_4_^+^, H^+^, and Pb(II) ions competed with each other for the adsorption sites on magnetite. When the solution pH value rose to 9.0 or higher, part of NH_4_^+^ was converted to free ammonia molecules, and the remaining NH_4_^+^, Pb(II) ions and lead-hydroxyl complexes competed for the adsorption sites on magnetite. This results in a lower removal efficiency of Pb(II) ions for the sample group compared to the control group, indicating that the presence of NH_3_-N in solution could potentially decrease the adsorption capacity of Pb(II) ions by forming a compete.

Typically, the effectiveness of heavy metal removal by an adsorbent tends to increase with rising pH. Once the pH exceeds the pH threshold for a specific heavy metal, the dominant adsorption process shifts toward hydrolysis and precipitation in solution. The pH threshold for lead ion is 7.6.^44^ Given the adsorption performance of the magnetite, the optimal solution pH for Pb(II) ion adsorption was identified to be 7.0.

#### Adsorption kinetics

**Figure 3:**
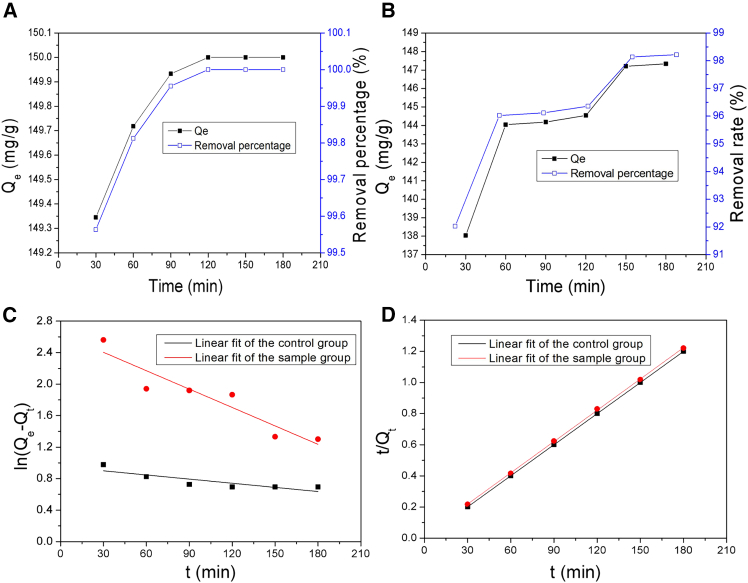
Effect of contact time on Pb(II) adsorption in two groups and the adsorption kinetics (A) The control group. (B) The sample group. (C) The pseudo-first order. (D) The pseudo-second order.

To obtain the data of adsorption kinetics for both groups, experiments were carried out at 25°C and pH 7.0, and 0.2 g of the MNs was added into 200 mL of a 150 mg/L Pb(II) ions solution. The mixture was stirred magnetically for 180 min. The outcomes are illustrated in Figure 3.

As shown in Figures 3A and 3B, the removal efficiency of Pb(II) ions improved significantly over time in both groups. In the control group (Figure 3A), the adsorption capacity of Pb(II) ions exhibited a significant rise during the initial 120 min, peaking at 150 mg/g. The corresponding removal percentage was 100%. This indicates that the adsorption process finished and reached to an equilibrium. In the sample group (Figure 3B), the adsorption capacity of Pb(II) ions exhibited a progressive enhancement from 0 to 150 min. Subsequently, it achieved an equilibrium within the range of 150–180 min. The final adsorption capacity and removal percentage were 147.33 mg/g and 98.22%, respectively.

**Figure 4:**
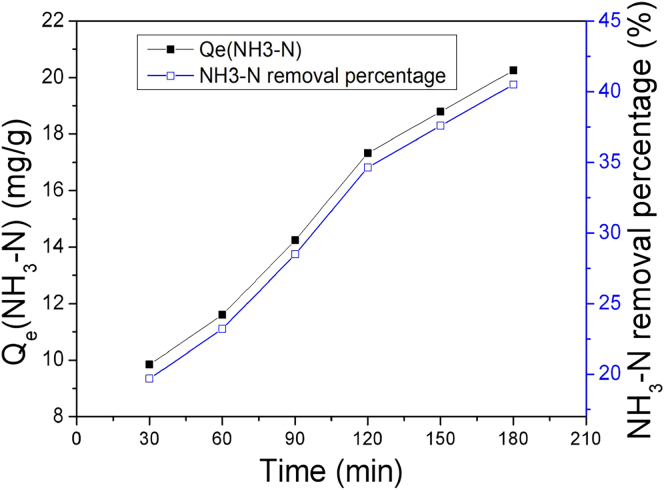
The adsorption of NH_3_-N by the MNs

As demonstrated in Figure 4, the curves show that NH_3_-N was indeed captured by the MNs in the sample group. Both the adsorption capacity and removal percentage increased with the contact time extending, and eventually reached 20.25 mg/g and 40.50%, respectively.

In order to evaluate the adsorption properties of the MNs, both the pseudo-first-order^45^ and pseudo-second-order^46^ kinetic models were applied to investigate the adsorption kinetics of Pb(II) ions in the experiments. The uniformity between the experimental data and model-predicted values was evaluated using the correlation coefficient R^2^. Each model is expressed as follows (Equations 4 and 5):

(Equation 4) $$\ln\left(Q_{e}-Q_{t}\right)=\ln Q_{e}-K_{1}t$$

(Equation 5) $$t/Q_{t}=1/\left(k_{2}{Q_{e}}^{2}\right)+t/Q_{e}$$

Here, Q_e_ (mg/g) and Q_t_ (mg/g) are the metal uptake per unit weight of adsorbent at equilibrium and at time t (min), respectively. K_1_ (min^−1^) and K_2_ (g/mg·min) are the rate constant of pseudo-first-order and pseudo-second-order, respectively. The slope and intercept values were used to determine K_1_ and K_2_. These parameters were calculated and listed in Table 2. The plot of ln(Q_e_-Q_t_) versus t and t/Q_t_ vs. t is given in Figures 3C and 3D, respectively.

**Table 2:** Pseudo-first-order and pseudo-second-order kinetics data of Pb(II) ion adsorption by the MNs

Adsorption conditions	Pseudo-first-order			Pseudo-second-order		
	K_1_/min^−1^	Q_e_/(mg/g)	R^2^	K_2_/(g/mg·min)	Q_e_/(mg/g)	R^2^
Control group	0.002	2.59	0.681	4.40 × 10^−2^	149.25	1.0
Sample group	0.008	14.01	0.853	2.50 × 10^−3^	149.25	0.999

Table 2 indicates that the correlation coefficients (R^2^) of the pseudo-second order were 1.0 for the control group and 0.999 for the sample group; both values exceed 0.99 and are significantly higher than that of pseudo-first-order model (0.681 for the control group and 0.853 for the sample group). These findings suggest that the pseudo-second order offers a better fit for describing the adsorption behaviors of Pb(II) ions in both groups, implying that the adsorption process of Pb(II) ions might be chemisorptions.^47^ Additionally, the pseudo-second order rate constants (K_2_) were 4.40 × 10^−3^ g/mg·min for the control group and 2.50 × 10^−3^ g/mg·min for the sample group, indicating that the adsorption rate constant of the control group was significantly greater than that of the sample group, which aligned with the result of removal efficiency of Pb(II) ions from the experimental data.

#### Adsorption isotherms

To further investigate the adsorption behavior of Pb(II) ions on the MNs, adsorption isotherms were conducted for both groups. At 25°C and pH 7.0, 0.2 g of MNs was added into 200 mL of Pb(II) ion solution, with concentrations ranging from 25 to 150 mg/L (i.e., 25, 50, 75, 100, 125, and 150 mg/L). The resulting data were analyzed employing both the Langmuir^48^ and Freundlich^49^ isotherm models, with the corresponding equations given as Equations 6 and 7, respectively.

Langmuir equation:

(Equation 6) $$1/Q_{e}=1/\left(bQ_{m}\right)\;*\;1/C_{e}+1/Q_{m}$$

Freundlich equation:

(Equation 7) $$\mathrm{Ln}\;Q_{e}=\mathrm{Ln}\;K_{f}+1/n*\mathrm{Ln}\;C_{e}$$

Here, Q_e_ is the mass of Pb(II) ions adsorbed per unit mass of magnetite in mg/g at equilibrium, Q_m_ is the maximum adsorption capacity (mg/g), and b is the Langmuir constant (L/mg) relating to adsorption energy. K_f_ is the binding energy constant reflecting affinity of the MNs to Pb(II) ions, and n is the Freundlich constant.

**Figure 5:**
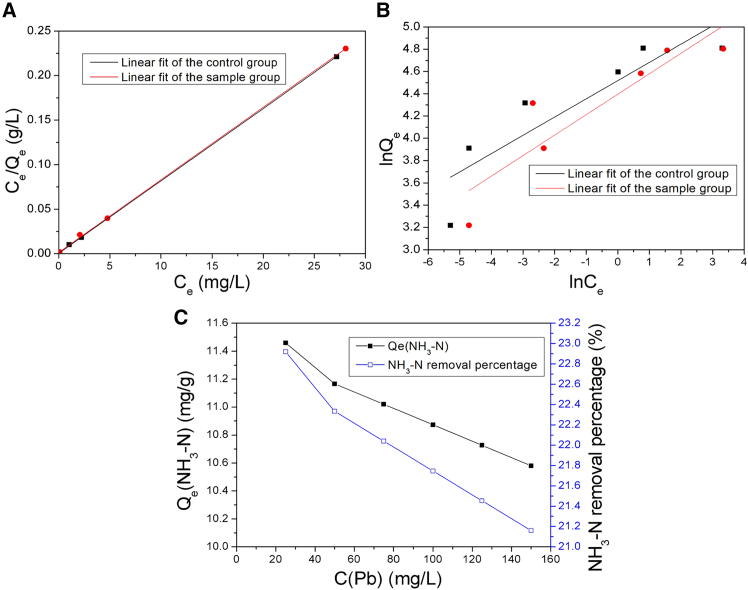
Adsorption isotherm fitting of Pb(II) ions of the two groups and removal efficiency of NH_3_-N in the sample group (A) Langmuir isotherm. (B) Freundlich isotherm. (C) Variation of NH_3_-N.

The linear fitting curves of the Langmuir and Freundlich models for both groups are depicted in Figures 5A and 5B, respectively. The corresponding fitted constants and regression coefficients (R^2^) are detailed in Table 3.

**Table 3:** The parameters of adsorption isotherms of Pb(II) ions

Adsorption conditions	Langmuir adsorption isotherms			Freundlich adsorption isotherms		
	q_m_/(mg/g)	b/(L/mg)	R^2^	n	K_f_ /(mg/g)·(L/mg)^1/n^	R^2^
Control group	123.15	1.57	0.9999	6.10	91.83	0.750
Sample group	122.55	6.09	0.9996	5.43	81.45	0.783

The Langmuir and Freundlich isotherms are referred to homogeneous and heterogeneous surface adsorption, respectively.^50^ Furthermore, the Langmuir isotherm elaborates a dominant ion exchange mechanism, while the Freundlich isotherm describes the adsorption-complexation reactions occurring during the adsorption process.^50^ As detailed in Table 3, a comparison of the correlation coefficients (R^2^) of two models reveals that the Langmuir isotherm model provides a more accurate representation of the experimental data than that of the Freundlich model. The maximum adsorption capacities were found to be 123.15 mg/g for the control group and 122.55 mg/g for the sample group, indicating that the adsorption of Pb(II) ions by the MNs follows monolayer uniform adsorption.^51^ Additionally, for the Freundlich isotherm model, *n* > 1 signifies favorable adsorption conditions.^52^^,^^53^ Therefore, the observed values of 6.10 for the control group and 5.43 for the sample group clearly indicate that the adsorption of Pb(II) ions by the MNs is a favorable adsorption and a highly effective adsorption process.

In the sample group, NH_3_-N was also removed by the MNs, as shown in Figure 5C. The adsorption capacity and removal percentage declined as the concentrations of Pb(II) ions increased. This suggested that the NH_3_-N occupied part of the adsorption sites of the MNs, which in turn reduced the adsorption capacity for Pb(II) ions. Meanwhile, it indicated that the affinity of magnetite for Pb(II) ions was higher than that of ammonia nitrogen.

#### Characterization of magnetic ferrite after the reaction

**Figure 6:**
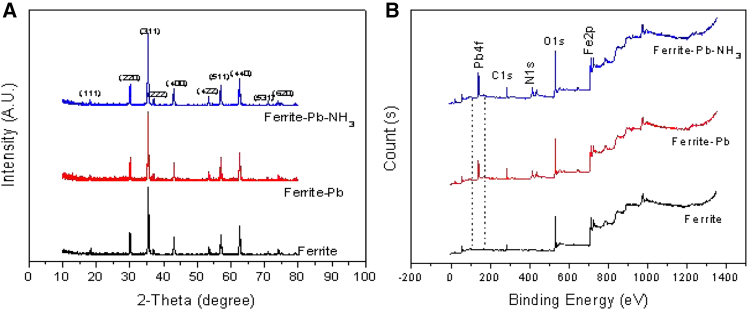
XRD and XPS analysis of the MNs before and after Pb(II) adsorption (A) XRD. (B) XPS.

To explore whether the crystal structure and phase composition of the MNs changed after Pb(II) ion and NH_3_-N adsorption, the comparative analyzes employing XRD and XPS were conducted on the MNs both before and after adsorption. The findings are presented in Figure 6.

The crystal structures of the MNs, both before and after Pb(II) ion and N adsorption, were characterized by XRD (Figure 6A). The XRD spectrum of the MNs after Pb(II) adsorption (for both control and sample groups) showed no significant changes compared to the pre-adsorption state. The 11 distinct reflection peaks observed were well aligned with the cubic inverse spinel structure of the Fe_3_O_4_ (JCPDS card no. 79-0416), confirming that the crystal structure of the MNs remained unaltered following Pb(II) ion adsorption.^54^ XPS was also employed to examine the oxidation state on surface of the MNs before and after Pb(II) ion and N adsorption (Figure 6B). As demonstrated by the XPS analysis, the distinct peaks at approximately 410.5 and 138.5 eV of the sample group confirmed the existence of Pb(II) ions and NH_3_-N on the surface of magnetite. The Fe 2p peaks at 710.7 eV agree with the reference data for magnetite,^55^ further confirming the identity of Fe_3_O_4_ and eliminating the other iron oxides. Additionally, the Pb4f peaks for both samples and the N 1s peak illustrated that Pb(II) ions and N were successfully adsorbed on the surface of the MNs without undergoing any redox reactions during the adsorption process.

Collectively, this study successfully synthesized a magnetic adsorbent with high adsorption efficiency and integrated into a process designed to remove Pb(II) ions from lead-ammonia wastewater. Characterizations of the LPSA and SEM illustrated that the magnetic particles had a spinel structure with an average particle size of 150 nm. XRD and XPS revealed that the synthesized particles were Fe_3_O_4_. The magnetite exhibited a saturation magnetization with the value of 82.30 emu/g, suggesting that it can be easily separated from wastewater under an external magnetic field. The prepared magnetite effectively removed Pb(II) ions from lead-ammonia wastewater. The effects of pH, contact time, and ammonia on Pb(II) ion adsorption were investigated, and the adsorption kinetics and adsorption isotherms for Pb(II) ion adsorption were also analyzed. The results indicated that Pb(II) ion adsorption follows the pseudo-second order and the Langmuir isotherm model. The maximum Pb(II) adsorption capacities were 123.15 and 122.55 mg/g for the control and sample group, respectively. Ammonia nitrogen in solution was also removed by the magnetite, so NH_3_-N that coexisted with Pb(II) ions in solution could reduce the adsorption capacity of Pb(II) ions. Characterizations of the magnetite before and after Pb(II) ion adsorption indicated that the crystalline structure of magnetite remained unaltered, confirming that Pb(II) ions and ammonia nitrogen were adsorbed on the surface without altering the structure of magnetite. Overall, the synthesized magnetite proved to be a cost-effective and environmentally friendly adsorbent for heavy metal removal from wastewater and had a broad prospect in the field of heavy metal complex wastewater treatment.

### Limitations of the study

This study illustrates that the magnetite can be prepared using a simple, cost-effective, and safe method and applied for removal of Pb(II) ions from complex wastewater. However, the analysis of Pb(II) ions adsorption mechanism was relatively weak, and the experimental data could be combined with microstructural characterization of magnetite to further elucidate the Pb(II) ion adsorption mechanism. Future studies should include a more detailed analysis of microstructural changes during the adsorption process, accompanied by some software calculations, to provide a more detailed explanation of the Pb(II) ion adsorption mechanism by magnetite.

## Resource availability

### Lead contact

Requests for further information and resources should be directed to the lead contact, Liu Fang (18767802532@163.com).

### Materials availability

This study did not generate new unique materials.

### Data and code availability

This article does not report original code. All data associated with this study are present in the article. Any additional information required to reanalyze the data reported in this article is available from the lead contact upon request.

## Acknowledgments

We thank the Fundamental Research Funds of Lishui University of Technology and the Natural Science Foundation of Zhejiang (TGS24E010003) for supporting this study.

## Author contributions

L.F., writing – original draft, writing – review & editing, and conceptualization; Z.K., conceptualization, supervision, methodology, and formal analysis.

## Declaration of interests

The authors declare no competing interests.

## STAR★Methods

### Key resources table

**Table:** 

REAGENT or RESOURCE	SOURCE	IDENTIFIER
**Chemicals, peptides, and recombinant proteins**		

Ferrous sulfate	Lishui Chemical Reagent Co.,Ltd.	CAS:17375-41-6
Sodium hydroxide	Lishui Chemical Reagent Co.,Ltd.	CAS:1310-73-2
Hydrochloric acid	Lishui Chemical Reagent Co.,Ltd.	CAS:7647-01-0
Lead nitrate	Lishui Chemical Reagent Co.,Ltd.	CAS:10099-74-8
Ethylenediamine tetraacetic acid	Lishui Chemical Reagent Co.,Ltd.	CAS:60-00-4
Sulfosalicylic acid	Lishui Chemical Reagent Co.,Ltd.	CAS:97-05-2
Hexamethylenetertramine	Lishui Chemical Reagent Co.,Ltd.	CAS:100-97-0
Magnetite	This paper	

**Software and algorithms**		

MDI Jade 6.5	Materials Data, Inc	https://www.icddchina.com
Avantage 5.52	Thermo Fisher Scientific	https://www.thermofisher.com
Original 8.5	OrigniLab	https://www.originlab.com

**Other**		

X-ray diffractometer	Beijing Purkinje General Instrument Co.,Ltd.	https://www.pgeneral.com
X-ray photoelectron spectroscopy	ThermoFisher-VG Scientific	https://www.thermo.com
Scanning electron microscope	Jeol Ltd.	https://www.jeol.com
Laser particle size analyzer	Beckman Coulter	https://www.beckmancoulter.com
Vibrating sample magnetometer	MicroSense	https://www.kla.com
Brunauer emmett teller	Micromeritics Instrument Corporation	https://www.micromeritics.com
Inductively coupled plasma-optical emission spectroscopy	Thermo Scientific	https://www.thermofisher.com

### Method details

#### Synthesis of the magnetite

The synthesis process was conducted at room temperature (25.0°C). Initially, 1.5 L of distilled water was added into a 2.0 L flat-bottom flask. Then flushed with nitrogen gas for 10 min and plugged with a rubber stopper to eliminate partly dissolved oxygen. Subsequently, 7.5 g of ferrous sulfate was introduced into the flask and thoroughly mixed using a magnetic stirrer. During this process, the pH of the solution was adjusted to 9.0 using 1.0 mol/L sodium hydroxide and 1.0 mol/L hydrochloric acid solutions. After the reaction was complete, the resulting black mixture was collected using a magnet, washed multiple times with ethanol and water, and then dried in a vacuum oven at 60°C.

### Quantification and statistical analysis

#### Characterization and analysis

The crystal structure was analyzed using an MSAL-XD2 X-ray diffractometer (XRD) with Cu Kα radiation (λ = 0.1541 nm) over a 2θ range of 10°–80°, operated at 30 kV and 30 mA. The surface composition was elucidated using X-ray photoelectron spectroscopy (XPS). The morphology and particle size were examined with a scanning electron microscope (SEM) at an electron acceleration voltage of 20 kV and a laser particle size analyzer (LPSA). The magnetic properties was assessed by a vibrating sample magnetometer (VSM). The specific surface area was measured by the Brunauer-Emmett-Teller (BET)employing N_2_ adsorption/desorption. The Fe^2+^/Fe^3+^ ratio was determined via complexometric titration.

#### Adsorption experiment

The stock solution of Pb(II) ions (1000 mg/L) was prepared by dissolving Pb(NO_3_)_2_ in double-distilled water, then was diluted to various concentrations ranging from 25 mg/L to 150 mg/L for use in the experiments. The experimental design included two groups of Pb(II) solutions: a control group without NH_3_-N and a sample group containing 50 mg/L NH_3_-N.In each experiment, a predetermined amount of magnetite particles was added to a 250 mL flask containing a specific concentration of Pb(II) ions. The flasks were sealed with rubber stoppers and stirred continuously during the reaction. The pH of the reaction mixture was adjusted using 0.1 M HCl and 0.1 M NaOH solutions.After the reaction, The reaction pH were adjusted using 0.1 M HCl and 0.1 M NaOH. After reaction, Pb(II) ions in supernatant solution was measured by a inductively coupled plasma-optical emission spectroscopy (ICP-OES) as the residual concentration (C_t_), and the removal rate (η) and the adsorption capacity (Q_e_) were calculated by the following equations: where C_o_ and C_e_ were the initial and the equilibrium concentrations of Pb(II) ion (mg/L), respectively, V is the volume of Pb(II)ion solution (L) and m is the weight of the ferrite used, and η is the removal rate of Pb(II)ion (%).
